# Potential for positive biodiversity outcomes under diet-driven land use change in Great Britain

**DOI:** 10.12688/wellcomeopenres.17698.1

**Published:** 2022-05-06

**Authors:** Henry Ferguson-Gow, Owen Nicholas, Charlotte Outhwaite, Rosie Green, Pauline Scheelbeek, Patricia Eustachio Colombo, Amber Wheeler, Anna Taylor, Alan D Dangour, Georgina Mace, Richard G Pearson

**Affiliations:** 1 Centre for Biodiversity and Environment Research, University College London, London, Greater London, WC1E 6BT, UK; 2 Department of Statistical Science, University College London, London, Greater London, WC1E 6BT, UK; 3 Centre for Ecology and Hydrology, Wallingford, Oxfordshire, OX10 8BB, UK; 4 Centre on Climate Change and Planetary Health, London School of Hygiene & Tropical Medicine, London, Greater London, WC1E 7HT, UK; 5 Department of Global Public Health, Karolinska Institutet, Stockholm, Sweden; 6 The Food Foundation, London, Greater London, SW9 7QD, UK

**Keywords:** biodiversity, land use change, public health, conservation, diets, species distribution modeling

## Abstract

**Background:** A shift toward human diets that include more fruit and vegetables, and less meat is a potential pathway to improve public health and reduce food system-related greenhouse gas emissions. Associated changes in land use could include conversion of grazing land into horticulture, which makes more efficient use of land per unit of dietary energy and frees-up land for other uses.

**Methods:** Here we use Great Britain as a case study to estimate potential impacts on biodiversity from converting grazing land to a mixture of horticulture and natural land covers by fitting species distribution models for over 800 species, including pollinating insects and species of conservation priority.

**Results:** Across several land use scenarios that consider the current ratio of domestic fruit and vegetable production to imports, our statistical models suggest a potential for gains to biodiversity, including a tendency for more species to gain habitable area than to lose habitable area. Moreover, the models suggest that climate change impacts on biodiversity could be mitigated to a degree by land use changes associated with dietary shifts.

**Conclusions:** Our analysis demonstrates that options exist for changing agricultural land uses in a way that can generate win-win-win outcomes for biodiversity, adaptation to climate change and public health.

## Introduction

Replacement of animal-derived protein (meat and dairy products) with fruits and vegetables in human diets is associated with reduced mortality from cardiovascular disease and some forms of cancer ( McEvoy *et al*., 2012; Yip *et al*., 2019) and offers multiple environmental benefits, including reductions in greenhouse gas emissions, land use footprints and terrestrial pollutants ( Castiglione & Mazzocchi, 2019; Eustachio Colombo *et al*., 2021; Scheelbeek *et al*., 2020a; Willett *et al*., 2019). Transformation of food systems is thus a key component of global ( IPCC, 2019) and national ( Climate Change Committee, 2019) efforts to mitigate climate change and support sustainable development ( Willett *et al*., 2019). Moreover, global studies have identified the potential of food system transformations to reverse negative trends in terrestrial biodiversity ( Leclère *et al*., 2020) such as the homogenization of biotic communities ( Dornelas *et al*., 2014), the loss of species richness ( Newbold *et al*., 2015) and declining population sizes ( WWF, 2020). However, global-scale analyses are unable to address national-level policy debates that will be vital for realizing this potential. Here we use Great Britain as a case study to explore how biodiversity conservation, climate change mitigation and public health might be jointly enhanced within a national policy context.

In the UK, the Eatwell Guide provides national public-health based recommendations on the relative contribution of food groups to a healthy and balanced diet ( Public Health England, 2016). The guide promotes a shift towards more plant-based diets and specifies that adults should eat at least five portions of fruit and vegetables per day, yet current levels of consumption fall well short of this recommendation ( Public Health England, 2020). Recent analysis has suggested that adherence to the Eatwell Guide is associated with reduced mortality as well as a reduction in diet-related greenhouse gas emissions ( Castiglione & Mazzocchi, 2019; Scheelbeek *et al*., 2020a). If the UK population were to adhere to the Eatwell Guidelines then there would need to be an increase in the supply of fruit and vegetables, which could be achieved through increased domestic horticultural production, increased imports of fruits and vegetables, or by a combination of the two. The UK currently imports about 84% of fruits and 56% of vegetables consumed ( DEFRA, 2021a) and, although meeting increased demand by increasing imports would avoid impacts within the UK, there are several reasons to favour increased domestic production, including reducing reliance on supply from climate vulnerable countries, increasing UK food security and avoiding environmental impacts in other countries ( Scheelbeek *et al*., 2020b).

Compared to the production of plant-derived foods, the production of animal-derived foods requires more land per kcal dietary energy produced ( Pimentel & Pimentel, 2003; Poore & Nemecek, 2018). For instance, 85% of the farmland globally that provides food for the UK is used to rear animals, yet animal-derived products provide only 32% of dietary energy consumed in the UK; by contrast, the 15% of farmland used to grow plants for human consumption provides 68% of dietary energy ( de Ruiter *et al*., 2017). Replacing meat with fruits and vegetables on a per-kcal basis could therefore reduce the land use footprint of the average diet. Currently 71% of the UK’s land surface is used for agriculture ( DEFRA, 2021b), so changes to patterns of land use (i.e., the relative amount used for animal- vs. plant-derived foods) could have a significant impact on the amount of land used for agriculture, and the amount available for other uses. To illustrate this, we consider the following two dietary scenarios for Great Britain (GB) in which meat is substituted with fruit and vegetables on a per-kcal basis to reach a threshold of 400g of fruit and vegetables consumed daily (consistent with the 5-a-day guideline; Eustachio Colombo *et al*., 2021): (1) in a domestic production only (DO) scenario, all additional demand for vegetables is met by expanding horticulture in GB (i.e., additional vegetables come from varieties that can be produced in GB); and (2) in a domestic/import (DI) scenario, current domestic production/import ratios are maintained (i.e., additional consumption is enabled by a combination of increased domestic production and increased imports). In the DO scenario, domestic horticultural production would be required to increase by 334% and meat production to decrease by 23%, and in the DI scenario domestic horticulture production would be required to increase by 123% and meat production to decrease by 30%.

To translate these changes in production into land use footprints, we assume that changes in production come from expanding or contracting the land use footprint of that type of agriculture, such that a 1% change in production corresponds to a 1% change in the footprint of the appropriate land use type. The increase in production could also be achieved through intensification on existing agricultural land, but we do not consider intensification here so our approach gives a conservative estimate of the land that could be freed to benefit biodiversity (see Discussion). When the DO and DI scenarios are converted into changes in land area (Methods), we find that in both scenarios the contraction in meat production accounts for a larger land footprint than the increase in horticulture production, meaning that under both scenarios land is made available for alternative uses. To explore the potential impacts of these shifts in land use on biodiversity, we examined cases whereby land taken out of meat production is first converted to horticultural production to cover the required increase in vegetables, and then the remaining land is converted to natural land covers. We did this for the DO and DI scenarios ( Table 1) as well as for three example scenarios that are consistent with the maximum 30% reduction in grazing land in the DI scenario: 15% of grazing land replaced with horticulture and 15% with natural land; 10% of grazing land replaced with horticulture and 20% with natural land; and 5% of grazing land replaced with horticulture and 25% with natural land.

**Table 1.** Conversions of grazing land to horticulture and natural land cover for two scenarios of diet-driven land use change in Great Britain.

	Percent of grazing land converted	
Scenario	To horticulture	To natural land cover
Domestic only (DO)	5%	18%
Domestic/Import (DI)	3%	27%

Both scenarios reflect an increase in vegetable intake for the population of GB to 400g of fruit and vegetables per day with a corresponding calorie-for-calorie reduction in meat consumption. Grazing land includes the land cover types rough grazing, permanent grassland and temperate grassland; and natural land cover includes the land cover types natural woodland and natural grassland.

We assessed the likely impacts on biodiversity of scenarios of land use change by constructing Species Distribution Models (SDMs) for 814 species in 4km ^2^ grid cells across GB (Methods). Species were chosen based on their inclusion in two indicators used by the UK government to report on progress towards international conservation targets: pollinating insects and priority species ( JNCC, 2021). We used a two-step modelling process to construct our SDMs: first we estimated the climatic envelope for each species across Western Europe and used this to project climate suitability in GB ( Pearce-Higgins *et al*., 2015; Pearson *et al*., 2002); and second, we used Bayesian logistic regression to model species occurrence within climatically suitable areas of GB as a function of 24 land cover classes. We ran the SDMs under current climate and under a future mid-range climate scenario (RCP 6.0) to test whether land use changes could, in part, mitigate the impacts of climate change (Methods).

Since there are numerous ways that land use change could be allocated spatially across the landscape, we first explored the case where the change is applied locally within each cell proportional to the quantity of grazing land already present. We then explored the effect of alternative land use allocation patterns by calculating how much each 4km ^2^ cell would stand to benefit in changes to habitable areas from the grazing land to horticulture transition. Using the results of our models, we generated two allocation strategies: one in which cells where a transition to horticulture was most beneficial for biodiversity were converted first (a ‘Best’ strategy) and another in which cells where a transition to horticulture was least beneficial were converted first (‘Worst’ strategy). The purpose of these alternative allocation strategies was to understand how sensitive our results were to the choice of land use allocation. All analyses excluded areas considered unsuitable for agricultural land use change, such as national parks, peatlands and steep slopes.

## Methods

### Data sources

***Species occurrence records*.** We focus on the species defined by the UK Joint Nature Conservation Committee (JNCC) and represented within the priority species indicator (Indicator C4) and the pollinating insects indicator (Indicator D1c; see underlying data ( Ferguson-Gow *et al*., 2022)), bolstered by additional bee and hoverfly species. These two indicators are used by the UK Department for Environment, Food and Rural Affairs as indicators of the health of UK biodiversity. We obtained occurrence records underpinning these indicators, which are derived from various national recording schemes and societies ( Table 2), from the UK Biological Records Centre. These data are used to generate the UK biodiversity indicators, ensuring consistency between our analyses and UK biodiversity indicators. The data were cleaned to ensure that the taxonomy was consistent and spurious records were discarded. Species observations were first collated from the various national recording schemes. These observations, or biological records, are presence-only data usually collected by volunteers as part of the recording scheme or society and consist of information on what species was observed, where it was observed and when it was observed. Since these data are often collected opportunistically and do not follow a sampling protocol, the information associated with individual records can vary, particularly in terms of spatial and temporal resolution. As a result, the raw observations were standardized and only those with consistent spatial and temporal precision taken forward. Only those records where the date was known to the day and where the location of the record could be specified at the 1km x 1km precision were used. Only records from the year 1970 onwards were included since the number of records at the required spatial precision tends to be very low before this period. Only records from within the UK were retained (excluding data from the Channel Islands, the Republic of Ireland and the Isle of Man). Any records at a taxonomic level higher than species were removed. The remaining species lists were then checked by scheme organisers to ensure synonyms were accounted for and to aid in the determination of species aggregates where necessary (for example, if changes in taxonomy over the time period of interest had occurred). After completion of the checks, duplicate records were removed. More information on this process can be found in the ‘Data Standardisation’ section of Outhwaite *et al.* (2019). Due to data availability our species pool represents a subset of the C4 DEFRA indicator.

**Table 2.** Recording schemes and societies contributing data.

Aquatic Heteroptera Recording Scheme	Gelechiid Recording Scheme
Bees, Wasps and Ants Recording Society	Grasshoppers and Related Insects Recording Scheme
British Arachnological Society, Spider Recording Scheme	Ground Beetle Recording Scheme
British Bryological Society	Lacewings and Allies Recording Scheme
British Dragonfly Society	National Moth Recording Scheme
Dragonfly Recording Network	Riverfly Recording Scheme: Ephemeroptera
British Myriapod and Isopod Group; Centipede Recording Scheme	Riverfly Recording Scheme: Plecoptera
British Myriapod and Isopod Group; Millipede Recording Scheme	Riverfly Recording Scheme: Trichoptera
Chrysomelidae Recording Scheme	Soldier Beetles, Jewel Beetles and Glow-worms Recording Scheme
Conchological Society of Great Britain and Ireland	Soldierflies and Allies Recording Scheme
Dipterists Forum: Cranefly Recording Scheme	Terrestrial Heteroptera Recording Scheme - Plant Bugs and Allied Species
Dipterists Forum: Empididae, Hybotidae and Dolichopodidae Recording Scheme	Terrestrial Heteroptera Recording Scheme - Shield Bugs and Allied Species
Dipterists Forum: Fungus Gnat Recording Scheme	UK Ladybird Survey
Dipterists Forum: Hoverfly Recording Scheme	Weevil and Bark Beetle Recording Scheme

To account for the fact that the UK may experience novel climates in the future and to more accurately characterise the climatic envelope of our species pool we modelled the climatic envelope of each species across their range in Western Europe, rather than just in the UK. The study area we used was between -8.2 and 2.7 longitude and 50 and 60.1 latitude, excluding Ireland and Northern Ireland. We obtained European occurrence records for bumblebees from the STEP project ( Potts *et al*., 2011; Rasmont *et al*., 2015) and records for the remaining species from GBIF by querying the database for the species binomial and all known synonyms ( Derived dataset GBIF.org, 2022). The GBIF records were cleaned using the R package CoordinateCleaner (v2.0-2), which removes potentially spurious records (e.g. those clustered around museums or research stations; Zizka *et al*., 2019). We used default settings in CoordinateCleaner: maximum record age of 1980 and minimum precision of 2 x 2 km. Finally, we rarefied the data to ensure that each cell in our study area contained a maximum of one occurrence record per species. Eighteen species with no European records (either the data were unavailable, or the species does not occur on mainland Europe) were retained to maximise the size of our dataset. We considered a species with fewer than 40 records in Great Britain as having insufficient data and thus eliminated them from the analysis. This left us with 1070 species in our species pool (457 pollinating insects and 613 priority species).

We generated pseudoabsences for each species by randomly sampling points from cells that: a) did not contain an occurrence record for the species; and b) were not within one cell of a cell that contained an occurrence record for the species. To ensure that the pseudoabsences represented the study area fairly, our sampling was uniform across the study area and we sampled either 500 pseudoabsences or an equal number to the occurrence records, whichever was higher ( Barbet-Massin *et al*., 2012).

***Climate data*.** We used bioclimatic variables obtained from CHELSA ( Karger *et al*., 2017) to predict habitat suitability across the study area for our chosen species. We used mean annual temperature, isothermality, mean annual precipitation and precipitation of the wettest month as our predictors. These variables are broad measures of temperature and precipitation and, given the taxonomic breadth of our dataset, we considered that they would be the most generically powerful to predict suitability for the widest range of species.

For future climate projections we obtained monthly predictions of maximum temperature, minimum temperature and precipitation from four global circulation models (MIROC-ESM-CHEM, NorES1-M, IPSL-CM5A-LR, GFDL-ESM2M, HadGEM2-ES) for every month in the time period 1970–2060 from the bias-corrected ISIMIP input dataset ( Hempel *et al*., 2013; Warszawski *et al*., 2014). These data were then converted into annual bioclimatic variables for each of the years in the time period 1979–2060 using the R package dismo v1.3-5 ( Hijmans *et al*., 2021). These data are at a much lower spatial resolution than the 2 x 2 km grid we were working to, so we used the scaling factor method to downscale the projections using the data from the years 1979–2013 to estimate a baseline ( Fordham *et al*., 2011). We then calculated the mean bioclimatic variables for each decadal period for each GCM giving four projections per decade, and then generated a mean across all GCMs to give a single ensemble mean projection of bioclimatic variables for the 2050s.

***Land use data*.** We used a UK land cover dataset that divides the UK into 2 x 2 km cells and describes the proportion of each cell that falls into each of 24 different land use classes, as described in Bateman *et al*. (2013; see extended data ( Ferguson-Gow *et al*., 2022)). Because the proportions of land use classes in each cell add to 1, we used logistic regression models for occurrence with all land use proportions included, but no constant term.

We obtained data from Lovett *et al*. (2014) to characterise each cell in the study area as suitable or unsuitable for agriculture and prevented any changes to the land use in cells considered unsuitable. Approximately 42% of the 2 x 2 km cells in our study area were considered suitable for land use change.

***Conversion of production changes to land area*.** DO scenario: Current grazing land (52,127 cells) is reduced by 23%, leaving 40,138 cells as grazing and 11,989 cells to redistribute. Horticulture (currently 600 cells) increases by 334% = 2,604 cells, which as a percent of the total current grazing cells = 2604/52127 = 5%, and the number of cells left for natural land cover = 11,989 - 2,604 = 9,385 cells (18% of grazing land).

DI scenario: Current grazing land (52,127 cells) is reduced by 30%, leaving 36,489 cells as grazing and 15,638 cells to redistribute. Horticulture (currently 600 cells) increases by 123% = 1,338 cells, which as a percent of the total grazing cells = 1338/52127 = 2.6%, and the number of cells left for natural land cover = 15,638 - 1,338 = 14,300 cells (27.4% of grazing land).

### Species distribution models

We employed a two-stage modelling process in which we first estimate the climatic envelope of each species, and then second estimate the effects of land use on species occurrence.

***Modelling species climatic envelopes*.** The occurrence records and pseudo-absences were used to establish the climatic envelope of each species by constructing ensemble species distribution models using BIOCLIM, generalised linear models (GLM) and random forest algorithms as implemented in the R package dismo ( Hijmans *et al*., 2021). We selected these three algorithms due to their varying reliance on absence data, and ability to perform well across relatively small study areas and under a wide range of data conditions. Each model was evaluated using five-fold cross-validation; we calculated the area under the receiver-operator curve (AUC) for each round of validation, taking the mean AUC as a measure of model performance. Models with AUC < 0.6 were discarded. Any species that did not achieve AUC ≥ 0.6 for at least two of the fitted models was removed from the analysis. This resulted in the elimination of 256 species, and a final dataset of 814 species ( Ferguson-Gow *et al*., 2022). For each model we generated a presence/absence prediction by identifying the threshold that maximises the sum of specificity and sensitivity ( Liu *et al*., 2013). Finally, we produced an ensemble prediction of presence or absence across the study area by combining the three models using a majority consensus rule: if two-thirds of the models predicted presence in a cell then that cell is present in the ensemble. This is a simple approach that accounts for the fact that the output of different algorithms may not be directly comparable ( Marmion *et al*., 2009). This process was repeated for each combination of relative concentration pathway and decade to generate predictions of each species climatic envelope under potential future conditions.

***Modelling species response to land use*.** Land use modelling was performed using the same GB occurrence records as we used for the climatic envelope modelling. Pseudoabsences were sampled from across GB from cells that were: (a) climatically suitable for the species, as predicted by our ensemble SDMs; (b) did not have an occurrence record in them; and (c) were not within one cell of a cell that contained an occurrence record. By doing this we are assuming that a cell that is climatically suitable but does not have an occurrence record is unsuitable due to the land cover configuration rather than the climatic conditions of the cell.

We pooled all presence and pseudoabsence records along with their associated values for the first seven principal components of the land cover data. The first seven PCs accounted for 95% of the variance in land cover. We then fitted a binomial mixed-effects model to a random 80% of these data, modelling presence/absence as a function of the land cover PCs, estimating a random slope and intercept for each species in the dataset. The remaining 20% of the data was set aside for model evaluation. From the resultant model we extracted the global parameter estimates and the species-specific parameter estimates and used these to estimate an AUC and threshold for each species, electing to use the threshold that maximises the sum of the sensitivity and specificity ( Liu *et al*., 2013). This process was repeated five times such that each datapoint was in the test dataset once (five-fold model evaluation) and for each species we took the mean AUC as a measure of the predictive power of the model for that species, and the mean threshold. As before, species that did not achieve an AUC of at least 0.6 were eliminated from the analysis.

We used Bayesian modelling for the presence, and pseudo-absence, of each remaining species expressed as a species-specific logistic function of proportion of land use in each class, with no constant term, resulting in 814 models. Experts specified prior distributions for the coefficients of each model declaring that the smallest absolute change in land use that could result in a shift from 50% to 75% (or equivalently 25%) chance of presence is one percent of the land use cell size. This prior was operationalised in the form of a product of 24 independent normal distributions centred at zero. We used two priors: one in which the standard deviation of the independent normals was 1, meaning that a priori for each land use class (measured in units of percentage) there was a 68% chance that the corresponding log odds was between -1 and 1; the other where the standard deviations were 1/6, meaning that a priori for all land use classes there was a 68% chance that the corresponding log odds were between -1 and 1.

These models were used to predict the probability of presence/absence in each cell for each species across the UK under scenarios of land use.

These models are naive to climate (other than the deliberate bias in the pseudo-absences) which means that when making predictions it is possible to predict a species to be present in cells that are outside of its climatic niche. In order to ensure that we are not making such predictions we apply the final step of using the species-specific climatic envelope as a mask to set all predicted values outside of the climatic envelope to zero after prediction. This same approach was used to make inferences of change under future climates by simply using the climatic envelope for a future climate scenario as a mask.

### Modelling land use change

We changed land use in three different ways. First, in each cell where land use could be modified we exchanged a proportion of its grazing land (permanent grassland, temporary grassland and rough grazing) to horticulture, and a further proportion of grazing land in equal amounts to the three natural covers (semi-natural grass, farm woodland and other woodland). The proportions were the same in all cells, and the result was an exchange of land use classes at the national level of the same proportions.

For the second and third strategies, we ordered cells by the expected improvement in average species occurrence per unit area of land use exchanged from grazing to horticulture, thus ordering cells by the benefit of exchanging grazing with horticulture in each cell. Then, for a given national proportion of exchange from grazing to horticulture we made this exchange in each cell, in order starting with the cell which we expected stood to benefit the most from such an exchange, until the appropriate national proportion was achieved (‘Best’ strategy). Also, for a given national proportion of exchange from grazing to natural covers, we worked with the same ordered list of cells but in reverse order, starting with exchange in the cell where transfer to horticulture was expected to be worst (‘Worst’ strategy).

### Modelling benefit to species

We used the models for each species in two ways. One was to forecast under a land use scenario the total probability for that species summed over the modifiable cells. We interpreted this figure as an expected area. Because there was uncertainty in each model’s coefficients, we carried forward that uncertainty in this calculation using linearisation. The other was to forecast, under a land use scenario, the chance of a 10% increase in the area of each species. Again, due to uncertainty in each model, we carried forward the uncertainty in the model in this calculation. We derived distributions of the number of species benefiting, and harmed, at the 10% level, again taking into account uncertainties in the models.

## Results

**Figure 1.**
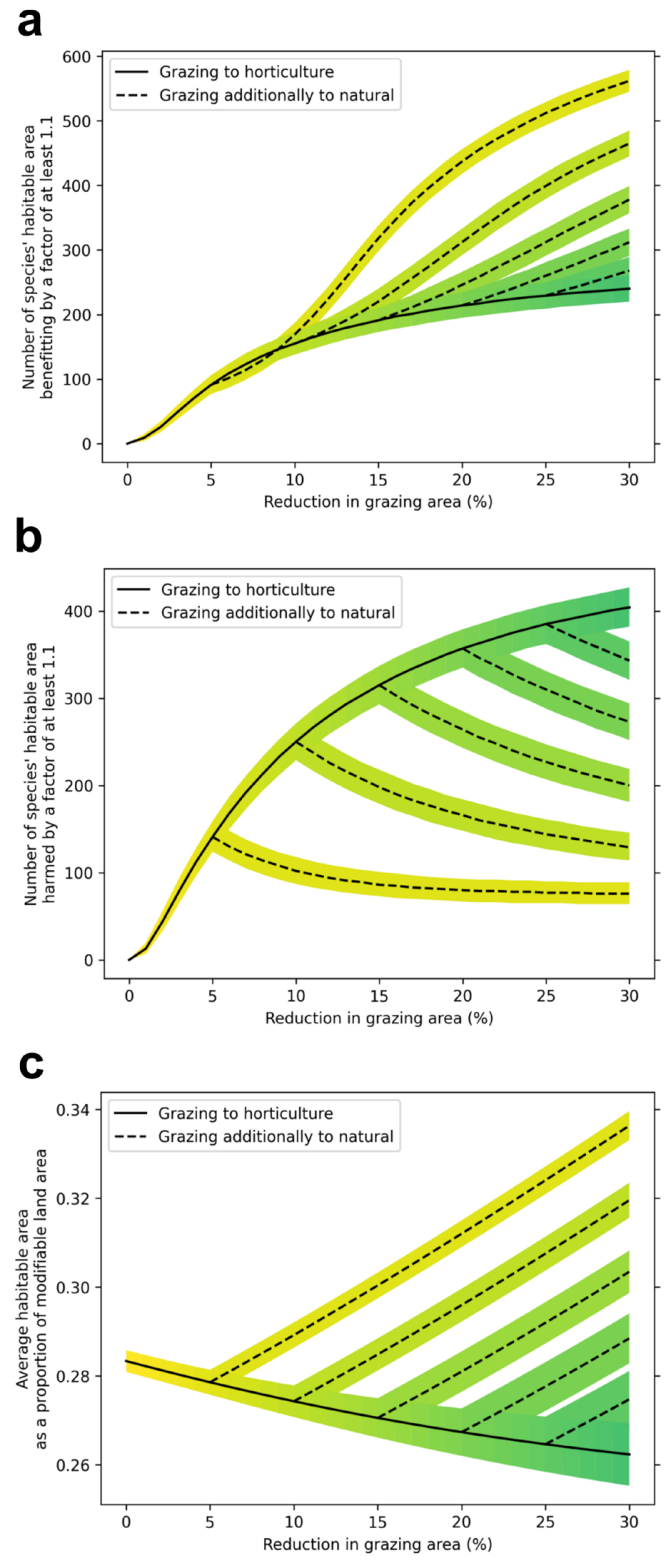
Projected species response to the replacement of grazing land with varying combinations of horticulture and natural land covers. **a**, The number of species that improve in habitable area by 10% or greater compared to their modelled habitable area under a no-change scenario. **b**, The number of species that decline in habitable area by 10% or greater compared to their modelled habitable area under a no-change scenario. **c**, The average habitable area as a percentage of modifiable land area. Solid black lines indicate a scenario where all grazing land removed from the landscape is replaced with horticulture. Dotted lines indicate scenarios where grazing land is replaced by horticulture until a certain percentage is reached (5, 10 and 15%) and then continues to be replaced by natural land covers.

We estimate that across all GB land area where agricultural land use could change, the average habitable area of 814 species is 28% and each 10% of grazing land transferred to horticulture is associated with 1 to 2% reduction in average habitable area; by comparison, each 10% of grazing land converted to natural land cover is associated with a 6% increase in average habitable area. Thus, on average conversion of grazing to horticulture results in a small loss of biodiversity, but this is outweighed by potential gains from converting the surplus grazing lands to natural cover ( Figure 1). As a result, all land conversion scenarios considered demonstrate the potential for gains to biodiversity, as measured by increases in average habitable area and more species gaining habitable areas by >10% than species losing habitable area by >10% ( Table 3). For each species losing >10% habitable area, we find that 6.3 (5.4–7.6) species will gain >10% habitable area under the DO scenario, and 9.8 (8.3–11.4) species will gain >10% under the DI scenario.

**Table 3.** Projected biodiversity responses to land use and climate change scenarios.

Grazing land conversion scenario	Average habitable area	# species >10% habitable area increase	# species >10% habitable area decrease
No change	0.283 (0.281–0.286)	-	-
15% to horticulture, 15% to natural	0.303 (0.299–0.308)	378 (357–399)	200 (181–219)
10% to horticulture, 20% to natural	0.320 (0.316–0.323)	465 (445–485)	129 (114–146)
5% to horticulture, 25% to natural	0.336 (0.333–0.340)	562 (545–579)	76 (64–89)
5% to horticulture, 18% to natural (DO)	0.319 (0.316–0.322)	485 (467–504)	78 (65–91)
3% to horticulture, 27% to natural (DI)	0.343 (0.340–0.346)	599 (583–615)	63 (52–74)
No land conversion, with climate change	0.205 (0.203–0.206)	23 (16–30)	649 (641–658)
5% to horticulture, 18% to natural (DO), with climate change	0.233 (0.231–0.236)	125 (112–138)	485 (469–500)
3% to horticulture, 27% to natural (DI), with climate change	0.253 (0.250–0.255)	213 (199–228)	406 (392–420)

DO = Domestic only production scenario; DI = Domestic/import production scenario. Average habitable area is presented as a percent of modifiable cells. Climate change scenario is RCP 6.0 for 2050s. Values in parentheses are 95% credibility intervals.

Including climate change (scenario RCP 6.0) in our models resulted in projections of strongly negative impacts on biodiversity, with average habitable area dropping from 28% to 21% in the absence of land use conversions ( Table 3). The number of species losing habitable area under climate change exceeds the number gaining habitable area: for each species gaining >10% habitable area, 4.1 (3.7–4.7) species will lose >10% under the DO scenario, and 2.0 (1.8–2.1) species will lose >10% under the DI scenario. Losses in habitable area due to climate change are larger than in any of our land use conversion scenarios, yet the models suggest that climate change impacts would be mitigated to some degree by the land use changes associated with a dietary shift from less meat to more vegetable consumption (e.g., average habitable area increases from 21% without land conversions to 23% under the DO scenario and 25% under the DI scenario; Table 3).

**Figure 2.**
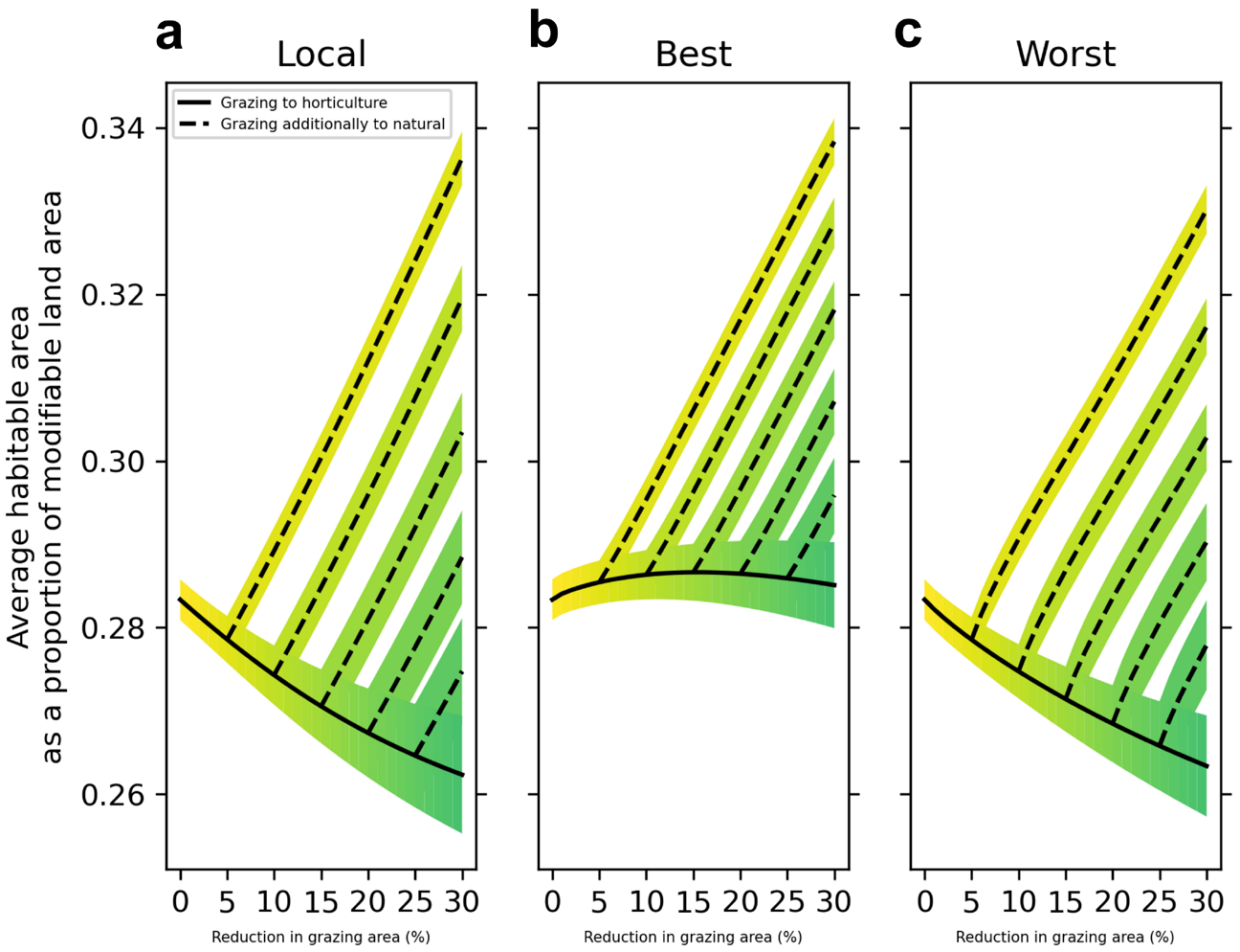
Change in average habitable area across all study species when grazing land is replaced with varying combinations of horticulture and natural land covers under three land use allocation strategies. **a**, Land use change is applied locally, proportionally to how much grazing land is present in each 4km ^2^ cell. **b and c,** We ordered cells according to our model's estimate, per unit area of transition from horticulture to grazing, of the benefit to the average habitable area of the 814 species. In **b** we allocated land use change to cells which had the best estimated benefit (‘Best’) and in **c** we allocated land use change to cells which had the worst estimated benefit (‘Worst’), to provide contrasting land use allocation scenarios. Solid black lines indicate a scenario where all grazing land removed from the landscape is replaced with horticulture. Dotted lines indicate scenarios where grazing land is replaced by horticulture until a certain percentage is reached (5, 10 and 15%) and then continues to be replaced by natural land covers.

In terms of how land use change is allocated spatially across the landscape, we find that prioritizing converting the locations that stand to benefit most from the grazing to horticulture transition results in the best outcomes for biodiversity ( Figure 2).

## Discussion

Following national dietary recommendations has previously been shown to have broad benefits for public health ( McEvoy *et al*., 2012; Scheelbeek *et al*., 2020a; Yip *et al*., 2019) and the environment ( Jarmul *et al*., 2020; Payne *et al*., 2016; Poore & Nemecek, 2018). Our analyses demonstrate that land use changes associated with healthier diets could also have benefits for biodiversity in GB and potentially increase resilience to climate change. The biodiversity benefits that our models suggest occur largely because the dietary energy equivalent replacement of meat with vegetables has the potential to result in the use of less land for agricultural production, thereby freeing up land for alternative uses. Our analysis repurposed agricultural land for natural habitats and found potentially large benefits for biodiversity, including lessening the potentially negative impacts of climate change on species by providing greater opportunity for range expansions ( Pearce-Higgins *et al*., 2015). This kind of ‘rewilding’ approach would of course be a policy choice and alternate policy decisions could see the land put to other uses (e.g., green energy production or house building) that would result in different impacts on biodiversity. We note that rewilding and habitat restoration are challenging and can take time to deliver benefits ( Perino *et al*., 2019; Pettorelli *et al*., 2018) but successful examples of rapid rehabilitation of nature exist ( Tree, 2017; Tree, 2018).

 Although we assumed a 1:1 relationship between the percent change in production and the associated land use footprint, intensification of horticulture could further increase the area of agricultural land that could be taken out of production, providing further potential for expansion of natural habitats or alternative land uses. Likewise, intensification of livestock grazing could provide potential for expansion of natural habitats, though with detrimental impacts on animal welfare. The benefits of horticultural intensification would trade-off against potential negative impacts such as soil erosion, community homogenisation, and nitrogenous pollution ( Tree, 2017) but sustainable intensification may be possible ( Dicks *et al*., 2019; Finch *et al*., 2019; Gunton *et al*., 2016). We also note that our study only considered change in meat consumption, rather than all animal source foods including dairy. Considering dairy as well would be expected to further increase the amount of land available for conversion to natural habitats.

 Our statistical biodiversity models provide first-pass estimates of species’ responses to land use and climate change, but these methods have several limitations, including that they do not account for interactions between species or the potential for rapid adaptation, nor do they estimate the dispersal capacity of species ( Pearson & Dawson, 2003). If an area is predicted to become more suitable for a species, it will still be necessary for the species to disperse through the landscape and colonize the new habitat, emphasising the need for connecting natural lands within resilient ecological networks ( Isaac *et al*., 2018). This, combined with our finding that the spatial allocation strategy affects biodiversity responses, highlights the challenge and opportunity for policy makers to ensure that future conversion of agricultural land to natural habitats and rewilding are managed in such a way as to maximize the benefits to biodiversity.

 Since our land conversion scenarios demonstrate larger benefits for biodiversity when the current ratio of domestic production to imports is maintained, versus when all additional demand is met by expanding horticulture in GB, it can be concluded that the best outcome for biodiversity in GB is to import more food and rely less on local production. However, our study did not look at the potential impact of diet changes beyond national borders. The current reliance of the UK on food imports compromises national food security, which is likely to be of particular importance as climate change alters patterns of trade, and also ‘offshores’ the environmental burden of UK diets to other countries, raising important questions about the equity of changes to national food systems ( Scheelbeek *et al*., 2020b). For example, increased demand for tropical fruits and exotic vegetables has led to rising imports of products from climate vulnerable countries ( Scheelbeek *et al*., 2020b). Further research is needed to better understand how global biodiversity impacts can be minimized in the context of international trade ( Ortiz *et al*., 2021).

Our study has implications for land management policies. For instance, the National Food Strategy for England sets targets for a 30% increase in fruit and vegetable consumption and a 30% reduction in meat consumption ( The National Food Strategy, 2021). Our results indicate that this can be achieved at the same time as improving biodiversity and mitigating some of the expected impacts on biodiversity of climate change, if some grazing land is allocated to natural land covers. However, to make these shifts in land-use, environmental land management schemes, such as the Sustainable Farming Incentive, Local Nature Recovery scheme and Landscape Recovery scheme ( DEFRA, 2022), will need to be designed to incentivise farmers accordingly.

## Conclusions

Our use of Great Britain as a case study helps improve understanding of how changing patterns of food consumption and associated land use within a state can be important in addressing environmental and health problems globally. By demonstrating the possibility of win-win-win outcomes, our analyses add to the growing evidence base that shows how reducing meat consumption in favour of increasingly plant-based diets can generate positive outcomes for public health, climate change mitigation, and biodiversity conservation.

## Data availability

### Underlying data

Zenodo: Underlying data for ‘Potential for positive biodiversity outcomes under diet-driven land use change in Great Britain’. https://doi.org/10.5281/zenodo.5950710 ( Ferguson-Gow *et al*., 2022).

This project contains the following underlying data:

- Data table 1. The 814 species that comprised the final dataset.

- Data table 2. The 24 land cover classes in the land cover dataset.
  (ferguson-gow_et_al_2022_extended_data.xlsx)

Zenodo: Underlying data for ‘Potential for positive biodiversity outcomes under diet-driven land use change in Great Britain’. https://doi.org/10.5281/zenodo.5939214 ( Derived dataset GBIF.org, 2022).

This project contains the following underlying data:

- Data table 1. GBIF filtered species occurrence records.
  (ferguson-gow_et_al_2022_gbif_data.xlsx)

Data are available under the terms of the Creative Commons Attribution 4.0 International license (CC-BY 4.0).

Species occurrence records, derived from national recording schemes and societies ( Table 2) were obtained from the UK Biological Records Centre. Anyone who wishes to gain access to these records can contact the Biological Records Centre ( brc@ceh.ac.uk) and make a request. European occurrence records for bumblebees obtained from the STEP project are available from the corresponding authors ( Potts *et al*., 2011; Rasmont *et al*., 2015). Bioclimatic variables obtained from CHELSA are available from: https://chelsa-climate.org/. The ISIMIP input data and data access instructions can be found here: https://www.isimip.org/gettingstarted/data-access/
