# Supplementary material for: Potential for positive biodiversity outcomes under diet-driven land use change in Great Britain
Source: Wellcome Open Res. 2024 Feb 15;7:147. Originally published 2022 May 6. [Version 2] doi: 10.12688/wellcomeopenres.17698.2 (PMC10948972; doi:10.12688/wellcomeopenres.17698.2)
Supplement: Peer review history

# Reviewer report: Reviewer response for version 2

**DOI:** 10.21956/wellcomeopenres.22968.r74620

**Published:** 2024-03-18

**Authors:** Patrícia Abrantes

**Affiliations:** 1 Centre of Geographical Studies and Associated Laboratory Terra, Institute of Geography and Spatial Planning, Universidade de Lisboa, Lisbon, Lisbon, Portugal

The article can be approved. The authors have made a great improvement

Is the work clearly and accurately presented and does it cite the current literature?

Partly

If applicable, is the statistical analysis and its interpretation appropriate?

Partly

Are all the source data underlying the results available to ensure full reproducibility?

Partly

Is the study design appropriate and is the work technically sound?

Yes

Are the conclusions drawn adequately supported by the results?

Partly

Are sufficient details of methods and analysis provided to allow replication by others?

Partly

Reviewer Expertise:

Land use systems, GIS and spatial analysis, Land use planning

I confirm that I have read this submission and believe that I have an appropriate level of expertise to confirm that it is of an acceptable scientific standard.

# Reviewer report: Reviewer response for version 2

**DOI:** 10.21956/wellcomeopenres.22968.r74619

**Published:** 2024-02-22

**Authors:** Mishel Unar-Munguía

**Affiliations:** 1 Instituto Nacional de Salud Pública, Morelos, Mexico

I have no further comments to the manuscript. The authors attended my comments and explained in detail the modeled scenarios. Thank you

Is the work clearly and accurately presented and does it cite the current literature?

Partly

If applicable, is the statistical analysis and its interpretation appropriate?

I cannot comment. A qualified statistician is required.

Are all the source data underlying the results available to ensure full reproducibility?

Yes

Is the study design appropriate and is the work technically sound?

Yes

Are the conclusions drawn adequately supported by the results?

Yes

Are sufficient details of methods and analysis provided to allow replication by others?

Partly

Reviewer Expertise:

Population Nutrition, Health and nutrition economics

I confirm that I have read this submission and believe that I have an appropriate level of expertise to confirm that it is of an acceptable scientific standard.

# Reviewer report: Reviewer response for version 1

**DOI:** 10.21956/wellcomeopenres.19582.r58484

**Published:** 2023-07-17

**Authors:** Mishel Unar-Munguía

**Affiliations:** 1 Instituto Nacional de Salud Pública, Morelos, Mexico

This is an innovative study that will add evidence of the benefits of land use change in favor of producing foods for a healthy and sustainable diet. Due to my area of expertise, my comments will focus more on diet and the selected scenarios.

 It will be useful to provide information on current mean consumption in GB by food groups and also mention intake recommendations provided by the EATWELL guidelines.

 With the information provided, I don't understand why the authors modeled a scenario where F&V should increase by 30% and meat should decrease by 30%.  Also, in terms of kcal, a 30% reduction in meat intake would require a greater increase in the consumption of F&V since the energy density of these food groups is different.

 From my point of view, there are no reasons explained to assume that reductions in meat should be substituted only with F&V. Why not include other plant-based food such as legumes and seeds?

 The authors mention that in the domestic model, all F&V are produced in GB assuming that only F&V that could be locally grown are considered. What are these F&V? How do these differ from the F&V that are consumed from imports? Does the type of F&V produce affect the type and amount of biodiversity?

 Regarding the grazing land conversion, the authors should explain more about the logic behind the scenarios. All these scenarios provide the same production/consumption of F&V and reduction of meat for the population. How, if the authors mention that intensity in the production was not modeled o increase? 

 Also, the time period modeled for land conversion is not clear. How many months/years should it take to convert grazing into horticulture land in this model?

 Finally, some policy implications from the conclusions should be provided in terms of how to align policies in GB to incentivize not only agriculture changes but also to incentivize the consumption of F&V in the population

Is the work clearly and accurately presented and does it cite the current literature?

Partly

If applicable, is the statistical analysis and its interpretation appropriate?

I cannot comment. A qualified statistician is required.

Are all the source data underlying the results available to ensure full reproducibility?

Yes

Is the study design appropriate and is the work technically sound?

Yes

Are the conclusions drawn adequately supported by the results?

Yes

Are sufficient details of methods and analysis provided to allow replication by others?

Partly

Reviewer Expertise:

Population Nutrition, Health and nutrition economics

I confirm that I have read this submission and believe that I have an appropriate level of expertise to confirm that it is of an acceptable scientific standard, however I have significant reservations, as outlined above.

# Response

**Published:** 2023-12-15

**Authors:** Henry Ferguson-Gow

**Affiliations:** University College London, UK

**Response to review 2**

**Reviewer’s original comments are followed by our responses in bold.**

 This is an innovative study that will add evidence of the benefits of land use change in favor of producing foods for a healthy and sustainable diet.

**Thank you.**

 Due to my area of expertise, my comments will focus more on diet and the selected scenarios.

 It will be useful to provide information on current mean consumption in GB by food groups and also mention intake recommendations provided by the EATWELL guidelines.

**We appreciate that this information will provide useful context for our study. We reference government statistics that give details about consumption levels and have now added to our manuscript a statement that summarises the average fruit and vegetable consumption, thus providing context as to how far away consumption is from the Eatwell target. The text now reads:**

**“In the UK, the Eatwell Guide provides national public-health based recommendations on the relative contribution of food groups to a healthy and balanced diet (** **Public Health England, 2016** **). The guide promotes a shift towards more plant-based diets and specifies that adults should eat at least five portions of fruit and vegetables per day. However, recent levels of fruit and vegetable consumption are around 344g per day, which falls short of the recommended 400g per day (** **Public Health England, 2020** **).”**

 With the information provided, I don't understand why the authors modeled a scenario where F&V should increase by 30% and meat should decrease by 30%.  Also, in terms of kcal, a 30% reduction in meat intake would require a greater increase in the consumption of F&V since the energy density of these food groups is different.

**We think the reviewer has misunderstood the scenarios we use. The reviewer is correct that reductions in meat intake will require greater increases in fruit and vegetables due to the different energy densities. This is, in fact, reflected in our scenarios. The following text describing the scenarios has been updated in Version 2 and we hope now provides clarity:**

**“… we consider two narrative dietary scenarios for Great Britain (GB) in which current average meat consumption is substituted with fruit and vegetables on a per-kcal basis to reach a threshold of 400g of fruit and vegetables consumed daily (consistent with the 5-a-day guideline). A number of previous studies have indicated that there would be benefits to both human health and greenhouse gas emissions from replacing meat with fruit and vegetables in UK diets, but most studies have not considered the origins of these foods or the potential co-benefits to biodiversity.** **The two scenarios were defined focusing on one important area of current environmental policy debate: whether consumption of fruit and vegetables produced in the United Kingdom should be prioritized over imported fruit and vegetable varieties to support national food security and resilience to climate change. The scenarios are described in detail in Eustachio Colombo *et al*., 2021 and are as follows** **: (1) in a domestic production only (DO) scenario, all additional demand for vegetables is met by expanding horticulture in GB (i.e., additional vegetables come from varieties that can be produced in GB); and (2) in a domestic/import (DI) scenario, current domestic production/import ratios are maintained (i.e., additional consumption is enabled by a combination of increased domestic production and increased imports). In the DO scenario, domestic horticultural production would be required to increase by 334% and meat production to decrease by 23%, and in the DI scenario domestic horticulture production would be required to increase by 123% and meat production to decrease by 30%.”**

 From my point of view, there are no reasons explained to assume that reductions in meat should be substituted only with F&V. Why not include other plant-based food such as legumes and seeds?

**Our analysis does, in fact, include legumes. We are sorry that wasn’t clearer in Version 1. We have included the vast majority of calories that would come from vegetables (including legumes) and note that the percent of diet represented by seeds is likely to remain very small. We have added the following text to the Discussion to clarify this issue:**

**“We made some necessary assumptions about dietary substitution in our analysis. These assumptions were substantiated by data from previous modeling studies indicating that vegetables (including legumes) are a plausible substitute for meat (Tiffin et al. 2011; Springman et al. 2018), as well as very recent analyses of food consumption trends in the United Kingdom (Environmental Audit Committee 2019). However, we cannot definitively determine that such substitutions would occur in practice and note the potential importance of novel plant-based foods in the future.”**

 The authors mention that in the domestic model, all F&V are produced in GB assuming that only F&V that could be locally grown are considered. What are these F&V? How do these differ from the F&V that are consumed from imports?

**These are fruit and vegetables that are already produced in the UK and have been identified as having potential for expansion. We cite Eustachio Colombo et al., 2021 as providing details; specifically, Supplementary Table 7 in that paper lists all fruits and vegetables produced in the UK, and Supplementary Table 4 lists fruits and vegetables consumed from imports.**

 Does the type of F&V produce affect the type and amount of biodiversity?

**This is an interesting question but we are not able with the data available to work out the impact ofo particular fruits and vegetables on biodiversity (e.g., we cannot estimate the specific impact of cucumber, cauliflower, or asparagus on particular species). Instead, our study is able to estimate the overall impact on biodiversity of a shift away from meat productions toward horticultural production (see results in Table 3).**

 Regarding the grazing land conversion, the authors should explain more about the logic behind the scenarios. All these scenarios provide the same production/consumption of F&V and reduction of meat for the population. How, if the authors mention that intensity in the production was not modeled to increase? 

**The land conversion scenarios that we developed assume that changes in production are achieved by expanding or contracting the land use footprint of that type of agriculture, rather than by changing the intensity. We explain this in the manuscript here:**

**“To translate these changes in production into land use footprints, we assume that changes in production come from expanding or contracting the land use footprint of that type of agriculture, such that a 1% change in production corresponds to a 1% change in the footprint of the appropriate land use type. The increase in production could also be achieved through intensification on existing agricultural land, but we do not consider intensification here so our approach gives a conservative estimate of the land that could be freed to benefit biodiversity (see Discussion).”**

**And also here:**

**“Although we assumed a 1:1 relationship between the percent change in production and the associated land use footprint, intensification of horticulture could further increase the area of agricultural land that could be taken out of production, providing further potential for expansion of natural habitats or alternative land uses. Likewise, intensification of livestock grazing could provide potential for expansion of natural habitats, though with detrimental impacts on animal welfare. The benefits of horticultural intensification would trade-off against potential negative impacts such as soil erosion, community homogenisation, and nitrogenous pollution (Tree, 2017) but sustainable intensification may be possible (Dicks et al., 2019; Finch et al., 2019; Gunton et al., 2016).”**

**We maintain that this approach is robust in providing a conservative estimate of the land that could be freed from dietary change (conservative in so far as the gains in land could be greater if intensification also occurred).**

 Also, the time period modeled for land conversion is not clear. How many months/years should it take to convert grazing into horticulture land in this model?

**We modelled biodiversity responses to land use change scenarios without specifying a time frame since assessing the factors that could affect this (e.g., policy options, economic drivers) is beyond the scope of our study. Stating a timeframe is unnecessary for our analyses, but the reviewer is correct that this is an aspect of the work that we haven’t explored and we therefore added the following to the Discussion:**

**“Our analyses did not specify a timeframe for realizing the different land use allocation scenarios, yet biodiversity responses are likely to be different depending on how rapidly land use change occurs. Exploring the temporal dynamics of how biodiversity will respond and the different policy decisions that could drive slow or fast change could be a fruitful avenue for further research.”**

 Finally, some policy implications from the conclusions should be provided in terms of how to align policies in GB to incentivize not only agriculture changes but also to incentivize the consumption of F&V in the population

**We see the work as highly policy relevant and conclude the manuscript with the following text (updated from Version 1):**

**“Our study has implications for land management policies. For instance, the 2021 independent review to inform a National Food Strategy for England suggested targets for a 30% increase in fruit and vegetable consumption and a 30% reduction in meat consumption (** **National food strategy for England, 2021** **). While these targets have not yet been adopted by the government’s official Food Strategy (** **Government food strategy 2022** **), our results indicate that they could be achieved at the same time as improving biodiversity and mitigating some of the expected impacts on biodiversity of climate change, if some grazing land was allocated to natural land covers. However, to make these shifts in land-use, environmental land management schemes, such as the Sustainable Farming Incentive, Local Nature Recovery scheme and Landscape Recovery scheme (** **DEFRA, 2022** **), will need to be designed to incentivise farmers accordingly. A particular challenge will be to achieve the best outcomes by developing policies that are spatially targeted (Bateman et al. 2013).”**

**However, while our study aims to empirically show the potential benefits of agricultural changes and diet shifts, it is beyond the scope of this manuscript to draw further conclusions about the policies that should be implemented to incentivize change. This will require detailed analysis of the policy landscape and pros and cons of alternative strategies, which is work we hope our study will inspire.**

# Reviewer report: Reviewer response for version 1

**DOI:** 10.21956/wellcomeopenres.19582.r56822

**Published:** 2023-05-19

**Authors:** Patrícia Abrantes

**Affiliations:** 1 Centre of Geographical Studies and Associated Laboratory Terra, Institute of Geography and Spatial Planning, Universidade de Lisboa, Lisbon, Lisbon, Portugal

The article is very interesting and seems innovative and pertinent in the current context, so it deserves to be published with the appropriate changes.

 From the structure point of view, the document should be improved. The introduction should focus on the problematic, the objectives and the state of the art, and the methodology referred to in the introduction should come later.

 In relation to the content, there is a lack of clear definition of the objectives of the article as well as the relevance of the data that justifies the conclusions, namely the outcomes for "biodiversity, adaptation to climate change and public health" ^1^.

 It lacks some theoretical reference about the scenarios ^2^ and the type of scenario should be framed (narrative, spatial-explicit...). I also think that there is a lack of contextualization about the evolution of diets and the type of food and consumption pattern (meat-based, seasonal, biological, local) that justifies the construction of the scenarios, as well as the contextualization of public policy (e.g. land use, health...).

 Regarding land use, what would be the advantage of using a finer spatial resolution? It is not explicit if the authors used a temporal analysis, i.e. two years of land use? If yes, then why didn't they use those transitions in the model? It should be better justified. A figure showing the workflow developed would helpful.

 As for the results and discussion. Since the results are not mapped it is difficult to ascertain what the impacts on the territory and on spatial policy. What kind of territories are most subject to change? The territories of urban sprawl and in the proximity of cities where more intensive agriculture is practiced, what changes would they bring? In terms of politics and public participation? Do farmers have an interest in changing? What about the role of consumers? Finally, what is the role of politics and what kind of policies should be favored? What is the relationship found between these results and public health?

Is the work clearly and accurately presented and does it cite the current literature?

Partly

If applicable, is the statistical analysis and its interpretation appropriate?

Partly

Are all the source data underlying the results available to ensure full reproducibility?

Partly

Is the study design appropriate and is the work technically sound?

Yes

Are the conclusions drawn adequately supported by the results?

Partly

Are sufficient details of methods and analysis provided to allow replication by others?

Partly

Reviewer Expertise:

Land use systems, GIS and spatial analysis, Land use planning

I confirm that I have read this submission and believe that I have an appropriate level of expertise to confirm that it is of an acceptable scientific standard, however I have significant reservations, as outlined above.

## References

[1] : Land use and climate change in the UK. *Land Use Policy* .2009;26: 10.1016/j.landusepol.2009.09.007 S160-S169 10.1016/j.landusepol.2009.09.007

[2] : Agricultural land fragmentation analysis in a peri-urban context: From the past into the future. *Ecological Indicators* .2019;97: 10.1016/j.ecolind.2018.10.025 380-388 10.1016/j.ecolind.2018.10.025

# Response

**Published:** 2023-12-15

**Authors:** Henry Ferguson-Gow

**Affiliations:** University College London, UK

**Response to review 1**

**Reviewer’s original comments are followed by our responses in bold.**

 The article is very interesting and seems innovative and pertinent in the current context, so it deserves to be published with the appropriate changes.

**Thank you.**

 From the structure point of view, the document should be improved. The introduction should focus on the problematic, the objectives and the state of the art, and the methodology referred to in the introduction should come later.

**We acknowledge that Version 1 had too much methodological detail in the Introduction. We have restructured the Introduction and Methods sections accordingly, moving descriptions of the conversion of production to land use footprint, the distribution modelling methods, and the spatial allocation approaches to Methods. We think that the division of information is now more appropriate and thank the referee for pointing this out.**

 In relation to the content, there is a lack of clear definition of the objectives of the article as well as the relevance of the data that justifies the conclusions, namely the outcomes for "biodiversity, adaptation to climate change and public health".

**As part of restructuring the Introduction (above), we now end the Introduction with the following concise statement of our central aim and the datasets used to achieve this:**

**“Our aim was to quantify the potential benefits to biodiversity that could come from diet-driven land use change. We did this by combining species occurrence records, climate change scenarios, and land use allocation strategies to model the potential distributions of 814 species in 4km ^2^ grid cells across GB.”**

 It lacks some theoretical reference about the scenarios and the type of scenario should be framed (narrative, spatial-explicit...). I also think that there is a lack of contextualization about the evolution of diets and the type of food and consumption pattern (meat-based, seasonal, biological, local) that justifies the construction of the scenarios, as well as the contextualization of public policy (e.g. land use, health...).

**Details about the dietary scenarios, including more context and much deeper justification of how and why they were constructed, are provided in our earlier paper (Eustachio Colombo et al., 2021). We had cited that paper in Version 1 but appreciate that some further details should be included in the revised paper. We have thus updated the key text describing the scenarios as follows:**

**“… we consider two narrative dietary scenarios for Great Britain (GB) in which current average meat consumption is substituted with fruit and vegetables on a per-kcal basis to reach a threshold of 400g of fruit and vegetables consumed daily (consistent with the 5-a-day guideline). A number of previous studies have indicated that there would be benefits to both human health and greenhouse gas emissions from replacing meat with fruit and vegetables in UK diets, but most studies have not considered the origins of these foods or the potential co-benefits to biodiversity.** **The two scenarios were defined focusing on one important area of current environmental policy debate: whether consumption of fruit and vegetables produced in the United Kingdom should be prioritized over imported fruit and vegetable varieties to support national food security and resilience to climate change. The scenarios are described in detail in Eustachio Colombo et al., 2021 and are as follows** **: (1) in a domestic production only (DO) scenario, all additional demand for vegetables is met by expanding horticulture in GB (i.e., additional vegetables come from varieties that can be produced in GB); and (2) in a domestic/import (DI) scenario, current domestic production/import ratios are maintained (i.e., additional consumption is enabled by a combination of increased domestic production and increased imports). In the DO scenario, domestic horticultural production would be required to increase by 334% and meat production to decrease by 23%, and in the DI scenario domestic horticulture production would be required to increase by 123% and meat production to decrease by 30%.”**

 Regarding land use, what would be the advantage of using a finer spatial resolution? It is not explicit if the authors used a temporal analysis, i.e. two years of land use? If yes, then why didn't they use those transitions in the model? It should be better justified. A figure showing the workflow developed would helpful.

**The land use dataset we use was developed for the UK National Ecosystem Assessment and has been widely used since (see Bateman et al. 2013 Science 341(6141): 45-50). Use of a land use dataset with finer spatial resolution is unlikely to result in qualitatively different findings since the data set we use captures the proportions of different land use classes within each 2x2 km cell. We do not refer to temporal land use analysis because we did not use multiple years of land use data; instead, our analyses are agnostic as to the timeframes involved – aside from the climate change scenarios (which are for the 2050s), we modelled biodiversity responses to land use change scenarios without specifying a time frame. Stating a timeframe is unnecessary for our analyses, and would not be useful since different policies would realize the scenarios over different time frames; however, the reviewer is correct that this is an aspect of the work that we haven’t explored and we therefore added the following to the Discussion:**

**“Our analyses did not specify a timeframe for realizing the different land use allocation scenarios, yet biodiversity responses are likely to be different depending on how rapidly land use change occurs. Exploring the temporal dynamics of how biodiversity will respond and the different policy decisions that could drive slow or fast change could be a fruitful avenue for further research.”**

**We think that the revised Methods section now better describes the approach taken and therefore do not see a workflow figure as necessary.**

 As for the results and discussion. Since the results are not mapped it is difficult to ascertain what the impacts on the territory and on spatial policy. What kind of territories are most subject to change? The territories of urban sprawl and in the proximity of cities where more intensive agriculture is practiced, what changes would they bring? In terms of politics and public participation? Do farmers have an interest in changing? What about the role of consumers? Finally, what is the role of politics and what kind of policies should be favored? What is the relationship found between these results and public health?

**The challenge to map our outputs is insightful. We have now done this and present the results in a new figure (Figure 3). Specifically, we mapped the distribution of land use changes under the three allocation strategies (local, best, worst) for the Domestic Only scenario. This has revealed an important new result: that the spatial patterns for alternative land allocation strategies are very different, which raises an important challenge for policy-makers to design policies that are spatially targeted. We have now added the following text to the manuscript to reflect this new analysis:**

**[Results:] “Mapping the land conversions derived from alternative allocation strategies (Figure 3) shows remarkably different spatial patterns. Local allocation spreads the land use changes relatively evenly across the landscape, whereas Best and Worst strategies concentrate change in more restricted areas. Of particular note are the somewhat opposing allocations between the Best and Worst scenarios, with the Worst strategy primarily allocating increased horticulture in England and the Best strategy allocating in Scotland and north Wales, and the Worst scenario primarily allocating new natural land covers in Scotland and north Wales, while the Best scenario allocates mostly in England.”**

**[Discussion:] “A particular challenge will be to achieve the best outcomes by developing policies that are spatially targeted (Bateman et al. 2013).”**

**The reviewer additionally raises some important questions about policy implications and incentivising farmers. We conclude the paper with the following text (updated from Version 1):**

**“Our study has implications for land management policies. For instance, the 2021 independent review to inform a National Food Strategy for England suggested targets for a 30% increase in fruit and vegetable consumption and a 30% reduction in meat consumption (** **National food strategy for England, 2021** **). While these targets have not yet been adopted by the government’s official Food Strategy (** **Government food strategy 2022** **), our results indicate that they could be achieved at the same time as improving biodiversity and mitigating some of the expected impacts on biodiversity of climate change, if some grazing land was allocated to natural land covers. However, to make these shifts in land-use, environmental land management schemes, such as the Sustainable Farming Incentive, Local Nature Recovery scheme and Landscape Recovery scheme (** **DEFRA, 2022** **), will need to be designed to incentivise farmers accordingly. A particular challenge will be to achieve the best outcomes by developing policies that are spatially targeted (Bateman et al. 2013).”**

**However, adding further discussion of the implications for policies and ways to incentivise farmers would require detailed policy analysis that is beyond the focus of this paper. Our central aim was to quantify the potential benefits to biodiversity that could come from diet-driven land use change, thus providing an empirical basis on which future policy work (addressing some of the questions raised by the reviewer) could build.**
